# Advances in the treatment of Hodgkin lymphoma: Current and future approaches

**DOI:** 10.3389/fonc.2023.1067289

**Published:** 2023-03-03

**Authors:** Fauzia Ullah, Danai Dima, Najiullah Omar, Olisaemeka Ogbue, Sairah Ahmed

**Affiliations:** ^1^ Department of Translational Hematology and Oncology Research, Cleveland Clinic Foundation, Cleveland, OH, United States; ^2^ Department of Hematology and Medical Oncology, Taussig Cancer Institute, Cleveland Clinic Foundation, Cleveland, OH, United States; ^3^ Department of Lymphoma/Myeloma and Stem Cell Transplant & Cellular Therapy, The University of Texas MD Anderson Cancer Center, Houston, TX, United States

**Keywords:** Classical Hodgkin lymphoma (cHL), relapsed and refractory Hodgkin's lymphoma, chemoimmunotherapy, hematopoietic stem cell transplant, chimeric antigen receptor (CAR) T-cell therapy

## Abstract

Hodgkin lymphoma (HL) is a rare type of lymphoma with unique histologic, immunophenotypic, and clinical features. It represents approximately one-tenth of lymphomas diagnosed in the United States and consists of two subtypes: classical Hodgkin’s lymphoma (cHL), which accounts for majority of HL cases, and nodular lymphocyte predominant Hodgkin lymphoma represent approximately 5% of Hodgkin lymphoma cases. From this point, we will be focusing on cHL in this review. In general, it is considered a highly curable disease with first-line chemotherapy with or without the addition of radiotherapy. However, there are patients with disease that relapses or fails to respond to frontline regimens and the standard treatment modality for chemo sensitive cHL is high dose chemotherapy followed by autologous hematopoietic stem cell transplant (AHSCT). In recent years, targeted immunotherapy has revolutionized the treatment of cHL while many novel agents are being explored in addition to chimeric antigen receptor (CAR) T-cell therapy which is also being investigated in clinical trials as a potential treatment option.

## 1 Introduction

Hodgkin lymphoma is derived primarily from B-cell lineage which consists of two subtypes, cHL and nodular lymphocyte predominant Hodgkin lymphoma. HL is a rare type of lymphoma and represents approximately 10% of the lymphomas in the UnitedStates with cHL accounting for nearly 95% of all HL cases (1), which is divided into nodular sclerosis, mixed cellularity, lymphocyte deplete variant, and lymphocyte rich variant (2). Reed-Sternberg cells are the pathognomonic malignant cell associated with cHL and drives continuous cell proliferation *via* NF-kB transcription factor expression (3, 4). cHL has a bimodal age distribution with a first peak around the age of 20-30 years and the second peak around the age 50-70 years which is more often associated with Epstein-Barr virus, and can occasionally occur in patients aged ≥75 years (5, 6). While cHL is considered highly curable with combination chemotherapy with or without the addition of radiotherapy (7), there are a small proportion of patients who do not respond or relapse after treatment with these therapies, and often high dose chemotherapy (HDC) and AHSCT can be curative in the 2^nd^ line setting (8–11).

For a select group of patients who have disease relapse after AHSCT or disease that is not chemo sensitive, allogeneic HSCT can provide a curative therapeutic option and the use of lower doses of chemo- or radiotherapy (reduced intensity conditioning, RIC) has significantly reduced toxicity while maintaining good outcomes (12–16). Prior to transplant, the goal of reducing disease burden is achieved with salvage chemotherapy, radiotherapy, or targeted agents (such as brentuximab vedotin or checkpoint inhibitors) and more frequently with a combination of chemotherapy agents or chemotherapy and immunotherapy. There are no randomized trials that directly compare salvage chemotherapy for relapsed HL. Response rates for salvage regimens in various phase II studies ranged from 60 to 85 percent (17). In this review, we will discuss the available novel therapeutic options and their efficacy and safety in the frontline and relapsed setting.

## 2 Frontline therapies in classical Hodgkin lymphoma

The therapeutic approach to cHL depends on stage at presentation, clinical prognostic factors and comorbidities. Staging is assessed by the Ann Arbor staging system (18). Treatment for early-stage (I-IIA) HL initially consisted of extended field radiation as the standard therapy. In the modern era, due to high relapse rates and significant long-term complications, extended field radiation therapy is no longer used (19). The current standard treatment for early-stage disease is either combination chemotherapy and involved-field radiation therapy (IFRT) or combination chemotherapy alone. The most widely used first-line chemotherapy regimen for cHL is a combination of doxorubicin, bleomycin, vinblastine, and dacarbazine (ABVD) (20). Moreover, Meyer et al. randomized 405 patients with early-stage disease to ABVD alone or subtotal nodal radiation therapy, with or without ABVD therapy (21). Patients who received subtotal nodal radiation therapy had a poorer overall survival (OS) (87% vs 94%), and on long term follow-up, higher rates of death from causes other than HL. A subsequent study randomized 1395 patients with unfavorable disease (large mediastinal masses, extra nodal disease, high erythrocytes sedimentation rate or ≥3 nodal sites) to ABVD for four cycles or standard doses of BEACOPP (bleomycin-etoposide-doxorubicin-cyclophosphamide-vincristine-procarbazine-prednisone) for four cycles, plus either 20 or 30 Gy IFRT (22). Treatment with ABVD plus 30 Gy was superior compared with 20 Gy, however, similar outcomes were seen between 20 and 30 Gy when used with BEACOPP. The German Hodgkin Study Group (GHSG) HD14 trial analyzed 1528 patients to ABVD for four cycles or escalated doses of BEACOPP for two cycles followed by ABVD for two cycles (2 + 2). All patients received 30 Gy IFRT. Freedom from treatment failure was superior with 2 + 2 regimen with a difference of 7.2% at 5 years, however, more acute toxicities were associated with this regimen (23). Based on multiple trials, early-stage favorable disease is generally treated with two cycles of ABVD followed by 20 Gy IFRT (24).

In patients with advanced stage (IIB-IV) disease, the MOPP regimen (nitrogen mustard, vincristine, procarbazine, and prednisone) was initially utilized for patients who progressed after radiation therapy and demonstrated an OS of 48% at 20 years (25). To improve efficacy and minimize toxicity, the ABVD regimen was developed. Multiple randomized studies have compared ABVD to MOPP and ABVD alternating with MOPP, the complete remission rate and freedom from progression was worse for patients receiving MOPP alone (26–29). The ABVD regimen showed superiority with less toxicity and is the most common treatment of choice for patients with advanced HL. The GHSG HD18 trial developed an escalated regimen for advanced-stage disease that consist of dose escalated BEACOPP which has shown higher response rates and progression free survival (PFS) in comparison to ABVD (30). Even though both regimens are associated with late toxic effects, such as secondary malignancies, infertility, cardiovascular disease and lung injury, these sequelae are more common with eBEACOPP (31–33). A randomized comparison of ABVD and BEACOPP of 331 patients with advanced stage HL reported the 7-year rate of freedom from first progression was 85% among patients treated with BEACOPP and 73% among patients treated with ABVD (P=0.004) (34). After completion of planned therapy, the 7-year rate of freedom from a second progression was reported as 88% in the BEACOPP group and 82% in the ABVD group (P=0.12), and the 7-year OS was 89% and 84%, respectively (P=0.39). Notably, severe adverse events were more common in the BEACOPP group than in the ABVD group. Recent trials have used positron emission tomography (PET) scans to identify advanced stage patients who may benefit from intensification or de-escalation of therapy, and the RATHL study is an example of this approach (35). In this study, 1214 patients with advanced HL had an interim PET scan following two cycles of ABVD therapy with those who had a negative scan (Deauville score (DS) 1-3) being randomized to receive ABVD or AVD (without bleomycin) for four more cycles. Patients with a positive PET scan (DS 4 or 5) proceeded to intensification therapy with either four cycles of BEACOPP or three cycles of escalated BEACOPP, followed by a repeat PET scan, if negative, patients received two further cycles of BEACOPP or one cycle of eBEACOPP. In patients with a negative interim PET scan, the 3-year PFS and OS rates in the ABVD group were 85.7% and 97.2% respectively, with similar outcomes in the AVD group with PFS and OS rates of 84.4% and 97.6%, respectively. The AVD group had less pulmonary toxicity. In patients with a positive interim PET scan, intensification of therapy to BEACOPP resulted in 3-year PFS rate of 67.5% and OS rate of 87.8%, suggesting that strategy of response-adapted therapy in HL improves outcomes.

While intensification of therapy to improve efficacy and safety in patients with advanced stage HL has been studied in the past, a more recent approach has been to add novel agents to standard chemotherapy regimens in the frontline setting, include brentuximab vedotin (BV) and PD-1 blocking antibodies. BV is an antibody-drug conjugate targeting CD30 expressed on the cHL cell surface and can be used in combination with standard regimen. The binding of BV to CD30 on the tumor cell membrane triggers a cascade of events which results in apoptotic death of the CD30-expressing cell (36). Checkpoint inhibitors exploit the well-known genetic alterations at 9p24.1 in cHL that leads to the overexpression of the ligands of programmed death-1 (PDL-1), consequently, PD-1 inhibitors have successfully been used to target the PD-1 axis (37–42).

The ECHELON-1 trial assessed the safety and efficacy of A+AVD (brentuximab vedotin, doxorubicin, vinblastine, and dacarbazine) versus ABVD in patients with stage III or IV cHL (43). In this trial, 1334 patients were randomized to receive up to 6 cycles of A+AVD (n=664) or ABVD (n=670). An updated analysis of the trial was presented at the 2022 ASCO annual meeting by the lead author Ansell et al. (44) reported the 6-year PFS of 82.3% with A+AVD and 74.5% with ABVD (hazard ratio [HR] 0.678, 95% CI 0.532-0.863). The 6-year OS rates were 93.9% and 89.4% with A+AVD vs ABVD, respectively, favoring A+AVD over ABVD (HR 0.590, 95% CI 0.396-0.879). Fewer secondary malignancies and more live births were reported with A+AVD. On the basis of these findings, the study recommends A+AVD over ABVD for patients with previously untreated stage III/IV cHL. A relatively recent phase II study of pembrolizumab, a humanized IgG4 monoclonal antibody targeting programmed death-1 (PD-1) protein evaluated outcomes in newly diagnosed cHL when combined with chemotherapy (45). Thirty patients (n=12 with early unfavorable stage and n=18 with advanced stage) were treated with 3 cycles of pembrolizumab monotherapy followed by doxorubicin, vinblastine, and dacarbazine (AVD) for 4 to 6 cycles. After cycle 3 of pembrolizumab monotherapy, 11 patients (37%) showed complete metabolic response (CMR), and 7 of 28 (25%) patients had >90% reduction in metabolic tumor volume on PET scans. All patients achieved CMR after 2 cycles of AVD and at a median follow-up of 22.5 months, there were no changes in terms of therapy, progressions or deaths. The most common adverse events were grade 1 rash and grade 2 infusion reactions. The result of this study suggests that pembrolizumab monotherapy followed by AVD was both effective and safe in patients with newly diagnosed cHL.

## 3 Management of relapse/refractory classical Hodgkin lymphoma

Approximately 20-30% of patients with cHL will be refractory to or relapse following frontline treatment (16). Salvage chemotherapy followed by HDC and AHSCT is the standard therapeutic option for patients with r/r cHL that is responsive to chemotherapy. Several studies have proven that HDC followed by AHSCT produce a better long-term disease-free survival and improved outcome than expected with conventional chemotherapy (8, 9, 46, 47). In a randomized trial by Schmitz et al, patients with relapsed Hodgkin’s disease were assigned to two cycles of Dexa-BEAM (dexamethasone and carmustine, etoposide, cytarabine, and melphalan) and either two further courses of Dexa-BEAM or high-dose BEAM and AHSCT in patients with chemosensitive disease. Freedom from treatment failure at 3 years was 55% for BEAM-AHSCT cohort compared with Dexa-BEAM (34%, P=0.019). OS did not differ significantly. Patients with disease progression after AHSCT have a poor prognosis with a median survival of 2.4 years (10, 48, 49). Relapse predictors post-AHSCT include relapse within 12 months of initial treatment, extra nodal disease, bulky disease, active disease at the time of transplant, primary refractory disease, and presence of B symptoms from lymphoma (50, 51).

### 3.1 Maintenance after AHSCT

Given the relatively high rate of post-AHSCT relapse in high risk patients, BV has been approved for maintenance after AHSCT in high risk r/r cHL (52–54). In the AETHERA phase III, double-blinded trial, 329 high risk HL post-AHSCT patients were randomized to receive 1.8 mg/kg of BV or placebo once every 3 weeks for up to 16 cycles starting 30 to 45 days post-transplant (55). Additional inclusion criteria included at least one of the following: primary refractory HL, relapsed HL with initial remission duration of less than 12 months, or extra-nodal involvement at the start of pre-transplantation salvage chemotherapy. Furthermore, patients had to have complete remission, partial remission or stable disease after pre-transplant salvage chemotherapy. Patients who received BV had a longer PFS than those who did not (median 42.9 months vs 24.1 months), and on a 5-year follow-up the PFS difference remained statistically different (59% vs 41%, respectively). While BV has been shown to be an effective maintenance option, it can cause significant peripheral neuropathy, which occurred in 56% of patients on trial in the BV arm in comparison to 16% of patients in the placebo arm. One limitation of this trial is the lack of universal PET scans prior to AHSCT, in fact one-third of patients did not have disease assessment by PET prior to transplant. It is possible that PET scanning done before AHSCT could have more accurately classified patient responses to salvage chemotherapy and the benefit of BV seemed to be diminished in patients who were PET-negative before AHSCT.

PD-1 inhibitors have also been evaluated as post-AHSCT maintenance therapy with the aim of improving rates of durable remission. Pembrolizumab and nivolumab are two anti-PD-1 antibodies currently approved in cHL patients with r/r disease (56, 57). In a multicohort phase II study of r/r cHL after AHSCT, 30 patients were enrolled to receive 8 cycles of pembrolizumab within 60 days of AHSCT, PFS at 18 months was 82% and OS 100%. Most adverse events (40%) were immune-related, grade 1 or II or higher (58). Similarly, nivolumab was evaluated as maintenance therapy post-AHSCT in high-risk r/r cHL (high risk defined as refractory disease, relapse <12 months, or relapse ≥12 months with extra nodal disease after frontline therapy). In a phase II single arm study, 37 patients were treated with nivolumab for 6 months, PFS was 92.1% at 6 months and the 12-month OS was 100%. The incidence of grade 3 or higher toxicity was 14% (59). Herrera et al. reported in abstract form on 59 patients who received BV and nivolumab maintenance post-AHSCT. Starting between day 30-75 after AHSCT, patients received 1.8 mg/kg of BV and 3 mg/kg nivolumab every 3 weeks for a planned 8 cycles. Forty-nine percent (29) of patients completed all 8 cycles with both drugs. Most common grade 2 or higher immune related adverse events included pneumonitis (12%), AST or ALT elevation (8%), hypothyroidism (5%), and rash (3%). The estimated 18-months PFS and OS for all patients were 95% and 98%, respectively (60). A summary of key studies (61–70) that evaluated the role of immune-checkpoint inhibitors and other novel therapies in cHL is shown in **Table 1**.

**Table 1:** Summary of key studies evaluating the role of novel therapies in relapsed and refractory cHL.

Study	Sample size	Regimen	Previous SCT	Median LOT	ORR	CRR	PFS	Median follow up (months)
Armand et al. (61)	31	Pembrolizumab	71% (autologous)	4+	65.0%	16.0%	69% at 24 wks, 46% at 52 wks	17
Chen et al. (62)	210	Pembrolizumab	61% (autologous)	4	69.0%	22.4%	63.4% at 9 months	10.1
Ansell et al. (63)	23	Nivolumab	78% (autologous)	3+	87.0%	17.0%	86% at 24 weeks	9.2
Younes et al. (64)	80	Nivolumab	100% (autologous)	4	66.3%	8.8%	76.9% at 6 months	8.6
Younes et al. (71)	129	Panobinostat	66% (autologous); 10% (allogeneic)	4	27.0%	4.0%	40% at 24 weeks	9.6
Armand et al. (72)	243	Nivolumab	100% (autologous)	4	69.0%	16.0%	Median 14.7 months	18
Herrera et al. (65)	62	Nivolumab + BV	NA	3	82.0%	61.0%	89% at 6 months	7.8
Song et al. (66)	70	Tislelizumab	18.6% (autologous)	3	87.1%	62.9%	74.5% at 9 months	9.8
Song et al. (67)	75	Camrelizumab	12.0% (autologous)	3	76.0%	28.0%	66.5% at 12 months	12.9
Shi et al. (68)	96	Sintilimab	19% (autologous)	3	80.4%	34%	77.6% at 6 months	10.5
Johnston et al. (69)	57	Everolimus	67% (autologous)	4	45.6%	8.8%	Median 7.3 months	Not available
Hamadani et al. (70)	60	Camidanlumab tesirine	49.3% (autologous); 10.4% (allogeneic)	5	73.1%	40.3%	Median 6.7 months	Not available

SCT, stem cell transplant; Median LOT, median line of treatment; ORR, overall response rate; CRR, complete response rate; PFS, progression free survival.

## 4 Salvage treatment modalities

### 4.1 BV-based salvage therapy

Given the success of BV in both advanced stage and r/r cHL, BV has been evaluated as salvage therapy after frontline therapy. Herrera et al. reported on a phase II study with 20 patients enrolled to a BV dose-escalation cohort that consisted of 1.8 mg/kg of BV intravenously every 3 weeks for two cycles (73). Patients with a complete response (CR) after two cycles received two additional cycles of BV at 1.8 mg/kg, while patients with stable disease or partial response (PR) were escalated to 2.4 mg/kg for two cycles. BV escalation was well tolerated however no patient was converted to CR from stable disease or PR. The overall response rate (ORR) was 75% and 43% of patients achieved CR. In total, 50% of patients proceeded directly to AHSCT (without post BV maintenance) with a 2-year PFS of 77%. After AHSCT, the 2-year PFS and OS were 67% and 93%, respectively. The 2-year PFS among patients in CR at the time of AHSCT was 71% compared with 54% in patients not in CR (P=0.12). Similarly, a non-randomized phase II trial evaluated the effect of weekly BV infusion on days 1, 8 and 15 for two 28-day cycles followed by a PET scan (74). Patients who achieved DS of 1 or 2 on PET scan proceeded directly to AHSCT and those with DS 3-5 on PET received two cycles of augmented ICE (isocyanide, carboplatin, etoposide) prior to consolidation with AHSCT. Twenty-seven percent of patients had a CR post-two cycles of BV and 69% of patients receiving augmented ICE subsequently attained a CR. For all patients treated with AHSCT, the 3-year OS and PFS were 95% and 82%, respectively.

The safety and efficacy of BV with bendamustine was evaluated in a phase I/II study of 55 patients with r/r HL (75). Patients received BV on day 1 and bendamustine on days 1 and 2 of a 21-day cycle for up to 6 cycles. Patients could continue on to AHSCT after cycle 2 and after a median of two cycles, the ORR was 93% with 73.6% of patients achieving CR. At a median follow up of 44.5 months, the 3-year OS and PFS were 92% and 60.3%, respectively (3-year PFS was 67.1% in patients who underwent AHSCT and 40.4% who did not undergo AHSCT) (76). A multicenter, phase I/II trial in patients with r/r HL was conducted by the Spanish Lymphoma Group (GELTAMO) (77). In total, 66 patients were assigned to a dose escalation BV with ESHAP (etoposide, methylprednisolone, cytarabine, and cisplatin) with ORR of 91%, CR 70% and the 30-month OS and PFS were 91% and 71%, respectively. Another phase I/II study evaluated BV in combination with dexamethasone, cisplatin, and cytarabine (BV-DHAP) for patients with r/r HL (78). Patients were treated with three cycles of therapy over 21 days, and PET was performed after three cycles with those attaining at least PR progressing to AHSCT. A total of 85% ultimately proceeded to AHSCT and the ORR and metabolic CR were 90% and 81%, respectively, with 2-year PFS of 74% and OS of 95%.

### 4.2 BV and checkpoint inhibitors-based salvage therapy

Brentuximab vedotin combined with nivolumab as a salvage therapy was evaluated in a phase I/II study in patients with r/r cHL (79). In this study, 93 patients were enrolled and of those 91 received the full study treatment with a median follow up of 34.3-months. The ORR for all treated patients was 85%, with 67% achieving CR, and the 3-year PFS was 91% and 3-year OS was 93% for patients who received an AHSCT after study treatment. Even though a high rate of infusion reactions (44%) was observed, the treatment was well tolerated overall. The safety and efficacy of ipilimumab, nivolumab, and BV were evaluated in a phase I study on 61 patients with r/r cHL treated with three different regimens (BV 1.8mg/kg with Ipe 1mg/kg and 3mg/kg; BV 1.2mg/kg and 1.8mg/kg combined with Nivo 3mg/kg; BV 1.2mg/kg and 1.8mg/kg with Nivo 3mg/kg and Ipi 1mg/kg) and ORR was 76%, 89% and 82% with CR rate of 57%, 61% and 73%, respectively (80). The median PFS was 1.2 years for BV-Ipi and not reached for BV-Nivo and BV-Nivo-Ipi. The most common adverse events were grade 3-4, included rash, gastritis, colitis, pancreatitis and arthritis. The phase II randomized portion of the trial is still ongoing (NCT01896999). In the KEYNOTE-204, a randomized multicenter phase III study, 151 patients were randomly assigned to pembrolizumab and 153 to BV (81). After 25 months, median PFS was 13.2 months for pembrolizumab versus 8.3 months for BV (HR=0.65, 95% CI 0.48-0.88). The most common grade 3-5 treatment related toxicity was pneumonitis (4% in the pembrolizumab group and 1% in the BV), neutropenia and peripheral neuropathy were more common in the BV group. The results suggest that pembrolizumab monotherapy is an effective and safe treatment option for patients with r/r cHL who relapse after AHSCT or who are ineligible for AHSCT.

### 4.3 Checkpoint inhibitors-based salvage therapy

The CHECKMATE-205 trial enrolled patients with disease that relapsed after AHSCT and underwent salvage therapy with nivolumab. ORR was 69% while median PFS and duration of response were 14.7 and 16.6 months, respectively (72). The results of this study led to the addition of checkpoint inhibitors (CPIs) with chemotherapy and subsequently a multicenter prospective trial explored nivolumab in combination with ICE (NICE) as first salvage in 39 patients with r/r HL who were enrolled and treated with nivolumab biweekly for up to 6 cycles (82). PET was performed after cycles 3 and 6, and patients who had not achieved a CR after cycle 6 went to receive two cycles of NICE. After nivolumab alone, the ORR was 81% with a 71% CR rate. Amongst the 9 patients who received NICE, the ORR was 100% with 89% of patients achieving a CR. The 2-year PFS and OS in all treated patients were 72% and 95%, respectively, while the 33 patients who bridged directly to AHSCT had a 2-year PFS of 94%.

Pembrolizumab with gemcitabine, vinorelbine, and liposomal doxorubicin (pembro-GVD) was studied in 39 patients in the second line setting for patients with r/r cHL (83). Those who achieved CR after 2-4 cycles proceeded to AHSCT. The ORR was 100% with 95% of patients achieving CR (92% after 2 cycles) and 95% proceeded to HDC/AHSCT. All patients who proceeded to transplant remained in remission at median post-transplant follow up of 12.5 months. A phase II study evaluated 42 AHSCT eligible patients with r/r cHL treated with pembrolizumab (PEM) added to ICE (84). Of the 42 patients enrolled, 37 patients were evaluable for efficacy and the CMR rate by PET following two cycles of PEM-ICE was 86.5% and the 24-month PFS was 88.2%. However, one patient died of cardiac arrest during stem cell collection, while another patient died due to acute respiratory failure secondary to engraftment syndrome early post-AHSCT.

Some studies have suggested that treatment with CPIs may re-sensitize patients to chemotherapy (85–87). Moreover, a multicenter study evaluated 81 heavily pretreated patients who progressed on CPIs were rechallenged, and sensitized patients to their subsequent treatment with ORR to post-CPI therapy of 62%, median PFS of 6.3 months and median OS of 21 months (87). There was no significant difference in OS. Subsequently, Calabretta et al. reported on 28 patients with r/r cHL, and of those, 26 (92%) were refractory to the last chemotherapy prior to CPIs (88). Following rechallenge with chemotherapy, 23 (82%) experienced a CR and 3 (11%) PR. Twenty-five patients proceeded to alloHSCT, and at a median follow-up of 21 months, median PFS and OS were not reached. A summary of key studies (89–92) that evaluated the role of immunotherapy and chemotherapy in the salvage setting in cHL is shown in **Table 2**.

**Table 2:** Summary of immunotherapy in addition to chemotherapy in the salvage setting.

Study	PFS	PET-Neg	Sample size	Regimen		
Santoro et al. (89)	59% @ 5 yrs. 77% for ASCT pts	75%	59	BEGEV Benda/gem/vinorelbine		
Moskowitz et al. (74)	73% @ 6 yr	83% 27% (BV alone)	65	BV-> augICE		Sequential BV and chemo
Lynch et al. (90)	80.4% @ 2 yr	74%	45	DD-BV-ICE		
LaCasce et al. (75)	62.6% @ 2 yr 69.8% for ASCT pts	74%	55	BV-benda		Combined BV and chemo
Stamatoullas et al. (91)	69% @ 1 yr	69%	39	ICE	plus BV	
Kersten et al. (78)	74% @ 2 yr	81%	61	DHAP		
Garcia-Sanz et al. (77)	71% @ 30 mo	70%	66	ESHAP		
Moskowitz et al. (92)	79% @ 2 yr	67%	91	BV-nivolumab		BV plus CPI
Mei et al. (82)	72% @ 2 yr for all 94% @ 2 yr for ASCT	91% 71% (Nivo alone)	43	Nivo-ICE		Combined CPI/chemo
Moskowitz et al. (83)	100% @ 1 yr post-ASCT	92%	39	Pembro-GVD		

PFS, progression free survival; PET, positron emission tomography; ASCT, autologous stem-cell transplant; BV, brentuximab vedotin; BEGEV, bendamustine, gemcitabine, vinorelbine; aug, augmented; ICE, ifosfamide, carboplatin, and etoposide; benda, bendamustine; DD, dose dense; DHAP, cisplatin, cytosine arabinoside, dexamethasone; ESHAP, etoposide, methylprednisolone, high-dose cytarabine, cisplatin; GVD, pembrolizumab plus gemcitabine, vinorelbine, and liposomal doxorubicin; CPI, checkpoint inhibitors.

## 5 Novel agents in r/r HL

Anti-PD-1 agents have been used extensively to treat cHL. Camrelizumab, a novel anti-PD-1 immune checkpoint inhibitor has been evaluated in a multicenter, phase II study in 75 patients with r/r cHL with median follow-up of 36.2 months (93). Median PFS was 22.5 months and 36-month OS rate was 82.7% (95% CI 72.0-89.5). The most common toxicity was grade 1 or 2 reactive capillary endothelial proliferation with spontaneous regression. A separate multicenter phase II study evaluated the efficacy and safety of GLS-010 or Fiberesima, an anti-PD-1, in Chinese patients with r/r cHL (94). The 12-month PFS and OS rate were 78% (95% CI 67.5-85.6) and 99% (95% CI 91.9-99.8), respectively. Treatment-related adverse events occurred in 92.9% of participants with grade III in 28.2% and most common were abnormal hepatic function, hyperuricemia and neutropenia. However, there is limited data on the long-term survival outcome of those patients after remission with immune-checkpoint inhibitors.

Treating r/r cHL remains challenging and multiple agents have been explored in this space, and one such agent are histone deacetylation (HDACs) inhibitors which function by blocking post-transcriptional histone modification in regulating gene transcription (95). Vorinostat (SAHA) inhibits STAT6 phosphorylation and transcription in HL cell lines which leads to decreased expression and secretion of Th2-type cytokines and chemokines (TARC and IL-5) and converse increase in Th1-type cytokines/chemokines (IP-10) (96). The Southwest Oncology Group (SWOG) reported on a phase II study of 25 patients with relapsed HL treated with single agent Vorinostat (97). While it was well tolerated, it only produced modest clinical activity, 1 patient (4%) achieving a PR and median PFS was 4.8 months. Mocetinostat (MGCD-0103) is a selective inhibitor of HDAC 1, 2, 3 (class I) and 11 (class IV) isoforms, which causes hyperacetylation of histones, and selectively induce apoptosis and cell cycle blockade in various cancer cell lines in a dose-dependent manner (98). The safety and efficacy of Mocetinostat was evaluated in a phase II study with an overall disease control rate of 34.8% and 25% for the 110-mg and 85-mg cohorts, respectively (99). After at least 2 cycles of therapy, 81% had a decrease in tumor measurements, however, 47% discontinued therapy due to disease progression (57% in the 85-mg cohort and 34% in 110-mg cohort). Panobinostat (LBH 589) is a pan-deacetylase inhibitor targeting epigenetic and non-epigenetic oncogenic pathways (100). Panobinostat was evaluated in a phase II study of 61 patients with r/r HL, and 53 patients completed two or more cycles of therapy and had at least one post-baseline imaging studies. Responses include one CR and 10 PR, with up to 92% decrease in tumor burden. The most common grade 3 and 4 adverse events were thrombocytopenia (77%), anemia (20%) and neutropenia (16%). In a similar study, 129 heavily pretreated patients were treated with Panobinostat with median time to response (TTR) of 2.3 months (71). Tumor reduction occurred in 74%, including 23% PR and 4% CR. Median PFS was 6.1 months and 1-year OS rate was 78%. Other HDAC inhibitors include Entinostat (SNDX-275) and ITF 2357 have shown encouraging clinical activity in r/r HL (101, 102).

Lymphocyte-activation gene 3 (LAG-3) is nearly always expressed in the tumor microenvironment of cHL and inhibitors of LAG-3 are now in clinical trials. *In vitro* studies have shown that anti-PD1 immunotherapy-resistant HL has CD8 lymphocytes depleted in microenvironment and overexpress the LAG-3 on CD4+ helper T lymphocytes (103). Timmerman et al. evaluated the safety and efficacy of favezelimab (MK-4280), a humanized IgG4 LAG-3 inhibitor, given with pembrolizumab every 3 weeks to 33 patients with r/r cHL refractory to anti-PD-1 therapy (104). At a median follow-up of 16.5 months, ORR for patients receiving favezelimab 800 mg was 31%, CR 7%, PR 24% and 66% of the responders had an anti-PD1-based regimen as most recent line of therapy. For all patients, median PFS and OS were 9-mo and 26-mo respectively, while 12-mo PFS and OS rates were 39% and 91% respectively. The most common adverse events were non-hematologic (hypothyroid, nausea, fatigue, etc). Another interesting target is the T-cell immunoglobulin and ITIM domains (TIGIT), which is an immune checkpoint receptor known to negatively regulate T cell functions and highly co-expressed with PD-1 on both CD4 and CD8 T cells in cancers (105). A study conducted by Annibali et al, 19/34 (56%) HL patients were TIGIT positive and of those, 16 (84%) were also PD-1 positive. Out of 15 TIGIT negative, only 4 (27%) were PD-1 positive, but (100%) were PD-L1 positive. Blockade of TIGIT with vibostolimab (MK-7684) has demonstrated antitumor activity in multiple pre-clinical tumor models. A multicohort, phase II study (106) is currently enrolling patients to evaluate the safety and efficacy of vibostolimab with pembrolizumab in patients with r/r cHL (NCT05005442). Camidanlumab tesirine is an anti-CD25 antibody drug conjugate that has been evaluated in r/r cHL. A recent phase I study explored camidanlumab tesirine in a highly pretreated patient population administered once every 3 weeks (107). Overall response rate was 71.4% and CR 48.6% in cHL cohort at 45 µg/kg camidanlumab tesirine with a median follow-up of 9.2 months. Toxicities were dose limiting with most common being rash, anemia, pyrexia and increased γ-glutamyl transferase. Five of 133 patients (3.8%) developed serious neurologic events of Guillain-Barré syndrome (GBS)/polyradiculopathy considered likely immune-related and at least possibly study-drug related but without correlation to dose. Treatment of relapse and refractory cHL remains an unmet need and further studies are needed to evaluate novel agents. A summary of recently completed or ongoing clinical trials of novel agents is shown in **Table 3**.

**Table 3:** Summary of clinical trials of novel agents in relapsed and refractory cHL.

Recently completed or ongoing clinical trials of novel agents (clinicaltrials.gov)			
Status	Study Title	Intervention	Identifier
Completed	Phase II Study of Oral Panobinostat in Adult Participants With Relapsed/Refractory Classical Hodgkin’s Lymphoma	Panobinostat	NCT00742027
Completed	RAD001 in Patients With Relapsed/Refractory Hodgkin Lymphoma That Has Progressed After High-dose Chemotherapy and Autologous Stem Cell Transplant and/or After Gemcitabine- or Vinorelbine- or Vinblastine-based Treatment.	Everolimus (RAD001)	NCT01022996
Ongoing	Magrolimab and Pembrolizumab in Relapsed or Refractory Classic Hodgkin Lymphoma	Magrolimab Pembrolizumab	NCT04788043
Active, not recruiting	Efficacy and Safety of Camidanlumab Tesirine (ADCT-301) in Patients With Relapsed or Refractory Hodgkin Lymphoma	Camidanlumab Tesirine	NCT04052997
Active, not recruiting	Nivolumab and Brentuximab Vedotin After Stem Cell Transplant in Treating Patients With Relapsed or Refractory High-Risk Classical Hodgkin Lymphoma	Nivolumab and brentuximab vedotin	NCT03057795
Ongoing	Chidamide+Decitabine+Camrelizumab Versus Decitabine+Camrelizumab in Anti-PD-1 Antibody Resistant Patients With Classical Hodgkin Lymphoma.	Chidamide+Decitabine+Camrelizumab vs. Decitabine+Camrelizumab	NCT04514081
Ongoing	Brentuximab Vedotin and Nivolumab With or Without Ipilimumab in Treating Patients With Relapsed or Refractory Hodgkin Lymphoma	Brentuximab Vedotin Ipilimumab Nivolumab	NCT01896999
Ongoing	Addition of Chidamide to the Combination Treatment of Decitabine Plus Camrelizumab in Combination Treatment Resistant/Relapsed Patients With Classical Hodgkin Lymphoma	Chidamide Camrelizumab Decitabine	NCT04233294
Ongoing	Safety and Preliminary Efficacy Assessment of AZD7789 in Patients With Relapsed or Refractory Classical Hodgkin Lymphoma	AZD7789	NCT05216835

## 6 Allogeneic HSCT in r/r cHL

The standard of care at first relapse after frontline therapy is HDC followed by AHSCT as part of salvage therapy. Patients who are eligible and achieve a complete metabolic response should proceed to AHSCT in second remission as the treatment related mortality is significantly lower when compared to alloHSCT. More recently with the availability of newer agents, the timing of alloHSCT is less clear. The decision considering alloHSCT should be individualized given the risk and potential benefit, however, in a selected group of patients it is potentially curative. We recommend alloHSCT be considered in all patients who have relapse of disease post AHSCT or are ineligible for AHSCT due to insufficient disease response with the caveat that patients must have responding lymphoma prior to proceeding to alloHSCT. The role of alloHSCT in patients with r/r cHL has been controversial due to high transplant-related mortality (TRM) as well as transplant related complications associated with acute and chronic graft-versus host disease (GVHD) (108). However, reduced-intensity conditioning (RIC) alloHSCT has shown a promising result with reduced TRM in patients with r/r cHL. In a clinical trial by Alvarez et al, 40 patients were treated with RIC alloHSCT demonstrated 2-year OS and PFS 48% ± 10% and 32% ± 10%, respectively (109). In chemo sensitive disease, the results were even better, 63% ± 12% and 55% ± 16%, respectively. In a phase II study (HDR-ALLO study), 92 patients with r/r cHL were treated with salvage chemotherapy followed by RIC alloHSCT, PFS rate was 48% at 1 year and 24% at 4 years (110). Patients who were allografted in complete remission, OS rate was 71% at 1 year and 43% at 4 years. The incidence of relapse rate was lower in patients with chronic GVHD. The addition of cyclophosphamide post-transplant showed better outcomes with 3-year OS, PFS, relapse rate and 1-year non-relapse mortality (NRM) rates of 63%, 59%, 21% and 20%, respectively (111). Post-transplant cyclophosphamide (PTCy)-based GVHD prophylaxis was associated with significant improvement in PFS and GRFS.

Patients with cHL with r/r disease may benefit from alloHSCT, but many lack a matched sibling donor (MSD). A study conducted by Ahmed et al. included 596 adult patients who received a first RIC alloHSCT using either a haplo-PTCy (n=139) or MSD/calcineurin inhibitor (CNI)-based (n=457) approach (112). On multivariate analysis, no significant difference was seen between haplo/PTCy and MSD/CNI-based approaches in terms of OS (HR 1.07, P=0.66) or PFS (HR 0.86, P=0.22). The haplo/PTCy platform was associated with higher risk of grades II to IV acute GVHD (OR 1.73, P=0.007) however, the risk of grades III to IV acute GVHD was not different between the 2 cohorts. The haplo/PTCy cohort had a significant reduction in chronic GVHD risk (HR 0.45, P < 0.001) and a significant reduction in relapse risk (HR 0.74, P= 0.03). A European study retrospectively compared the outcome of patients with HL who received PTCy-based haplo alloHSCT (n=98) vs HLA MSD (n=338) vs HLA-matched unrelated donor (MUD) (n=273) transplantation (113). The median follow-up of survivors was 29 months. Haplo alloHSCT was associated with a lower risk of chronic GVHD (26%) compared with MUD (41%, P=0.04). The 2-year cumulative incidence of relapse progression was 39%, 49%, and 32% in haplo, MSD and MUD, respectively. The two-year OS, PFS were 67% and 43% for haplo, 71% and 38% for MSD, and 62% and 45% for MUD, respectively. The rate of extensive chronic GVHD and relapse-free survival was significantly better for haplo (40%) compared with MSD (28%, P=0.049) and similar to MUD (38%, P=0.59). Based on the results of these studies there is support that haplo/PTCy alloHSCT in cHL results in survival comparable to traditional alloHSCT approaches with potentially better outcomes in terms of GVHD and relapse.

### 6.1 Tandem autologous-allogeneic transplantation

AHSCT is considered the standard treatment for patients with r/r HL (9). Risk factors that have been repeatedly found to be strong predictors of failure after AHSCT include HL refractory to frontline therapy and <12 months to first relapse (53, 55). For patients with high-risk of relapse after AHSCT, an alternative consolidation strategy with alloHSCT could be a potential option to improve their outcome (49, 50). The use of RIC has resulted in a significant reduction of the NRM but this strategy requires several months for the allogeneic effect to develop, thus in patients with an aggressive HL the disease might progress. In this setting, a tandem auto-RIC-SCT approach has the potential of providing effective cytoreduction to keep the lymphoma under control. A retrospective study conducted by Bento et al. analyzed 126 patients treated with tandem AHSCT- RIC-alloHSCT (114). The median number of lines prior to AHSCT was two (33% of the patients received >3 lines) and 41% were transplanted with active disease. The median follow-up was 44 months and 3-year PFS, OS, incidence of relapse, and NRM after the tandem were 53%, 73%, 34%, and 13%, respectively. A similar result was reported by Mariotti et al. in another study (115). The results suggest that this might be an effective treatment for a high-risk population.

### 6.2 PD-1 blockade prior to allogeneic HSCT

It is important to note that several studies have suggested PD-1 blockade prior to alloHSCT is associated with higher-than-normal rates of early transplant-related complications. Merryman et al. reported on a retrospective cohort of 209 cHL patients who received alloHSCT after PD-1 blockade with a median follow-up of 24 months (116). The 2-year GVHD and relapse-free survival (GRFS), PFS and OS were 47%, 69%, and 82%, respectively. The 180-day cumulative incidence (CI) of grade 3-4 acute GVHD was 15%, while the 2-year CI of chronic GVHD was 34%. Post-transplant cyclophosphamide (PTCy)-based GVHD prophylaxis was associated with significant improvements in PFS and GRFS. An international retrospective study reported on the outcomes of 39 patients with advanced lymphoma who received treatment with a PD-1 inhibitor at a median time of 62 days before alloHSCT (117). One-year OS and PFS rates were 89% and 76%, respectively, while the 1-year cumulative incidences of grade 2-4, grade 3-4, and grade 4 acute GVHD were 44%, 23%, and 13%, respectively. The incidence of grade 4 GVHD was higher than prior studies (13% vs 3% to 4%). In another multicenter retrospective analysis, 31 patients with relapse post-alloHSCT received anti-PD-1 with ORR of 77% (118). At last follow-up, there were 8 (26%) deaths related to new-onset GVHD after anti-PD1, 17 (55%) patients developed treatment-emergent GVHD after initiation of anti-PD1 and grade III-IV or severe chronic GVHD occurred in 9 patients. The results indicate that PD-1 blockade is highly efficacious but frequently complicated by rapid onset of severe and treatment-refractory GVHD. A similar finding was reported by Herbaux et al. analyzed 20 patients with HL relapsing after alloHSCT received anti-PD-1 (119). GVHD occurred in 6 patients (30%) and two patients died as a result of GVHD. The 1-year PFS was 58.2% and OS 78.7%. These results prompted a warning from the United States Food and Drug Administration and recommend caution about using PD-1 blockade in close proximity prior to alloHSCT.

## 7 CD30 CAR T-cells in Hodgkin lymphoma

Chimeric antigen receptor (CAR) T-cell against CD-30 is a directed novel cellular therapy where patient’s own immune cells are engineered ex vivo to recognize target cancer antigens. A phase I trial evaluated 18 heavily pretreated patients who received anti-CD30 CAR T-cells (120). This study used multiple combinations of drugs for conditioning. Patients received a conditioning regimen that included 3 forms: (I) FC (fludarabine, 3 daily doses of 25 mg/m^2^ + cyclophosphamide, at a total dose of 30 mg/kg) to deplete endogenous leukocytes; (II) GMC-like chemotherapy (gemcitabine 1 g + mustargen 10 mg + cyclophosphamide, at a total dose of 30 mg/kg) to inhibit the disease progression in a short time and eliminate endogenous leukocytes; and (III) PC (nab-paclitaxel 150 mg/m^2^ + cyclophosphamide 30 mg/kg) to deplete the stromal compartment and eliminate endogenous leukocytes. The individual absolute dose was administrated at the discretion of a physician according to treatment history and bone marrow tolerance. The ORR was 39% (all partial responders) with 28% of patients showing stable disease at two months and a median PFS of 6 months. All patients experienced Grade 1 or 2 febrile syndrome at the time of infusion that resolved without intervention. In another phase I/II trial of anti-CD30 CAR T-cells in r/r cHL, 41 heavily pretreated patients underwent lymphodepletion with varying regimens of bendamustine alone, bendamustine-fludarabine or cyclophosphamide-fludarabine (121). The ORR was 72% with 60% of patients attaining a CR. The one-year PFS and OS rates were 41% and 94%, respectively. Five patients had a CR more than a year out from infusion and more than a third showed durable response. The most common adverse event was cytopenia related to lymphodepletion with a favorable profile in terms of CART related toxicity. Subsequently, a phase II, multi-national trial (CHARIOT NCT04268706) which is ongoing, reported in abstract form on 15 patients with r/r cHL treated with CD30 CART (122). This was a heavily pre-treated patient population with 6 median prior therapies, with a range of 4 to 19. At the time of reporting, 12 patients had received CD30 CART infusion and were evaluable, the ORR at Day 42 was 100% (5/5), CR and PR rates were 80% (4) and 20% (1), respectively. The most common adverse events were hematologic and without significant CART associated toxicity such as cytokine release syndrome or neurotoxicity.

Voorhees and colleagues evaluated factors that were associated with PFS after anti-CD30 CAR T-cells therapy in r/r cHL (123). The tumor burden measured by using Flourine-18 fluorodeoxyglucose (F-FDG) PET imaging or metabolic tumor volume (MTV) showed a direct correlation between MTV and PFS. Patients with higher MTV (>60 ml) before CAR T-cells therapy and lymphodepletion had a lower 1-year PFS (14%) compared to those with a low MTV (58%) and interestingly, patients who responded to bridging therapy with a decrease in MTV had an improved 1-year PFS (40%) compared to those who showed no reduction in tumor burden post bridging therapy (1-year PFS 0%). However, bridging therapy was not associated with a significant difference in PFS neither the persistence of anti-CD30 CAR T-cells. In another study by Voorhees and colleagues, they evaluated the role of checkpoint inhibitors in patients with disease progression following CAR T-cell treatment and found a clinical benefit in all patients including those that had not responded to checkpoint inhibition prior to CAR T-cell therapy, presumably due to reprogramming and reactivating CAR T-cells that persisted after the initial infusion (124).

Other types of cellular therapy have also been explored with encouraging results of an ongoing trial with natural killer (NK) cells derived from umbilical cord blood (CB) and activated with a novel bispecific antibody known as AFM13 (targeting CD16A and CD30) demonstrated safety and efficacy in r/r CD30+ lymphoma (NCT04074746) (125). Patients received two cycles of fludarabine/cyclophosphamide followed by AFM13-precomplexed CB-NK cells (day 0) and 3 weekly intravenous infusion of AFM13. Eighteen patients completed both planned cycles and therapy was well tolerated. The ORR was 89% and at median follow-up of 6 months, PFS/OS across all dose levels were 52% and 81%, respectively. All patients treated at the recommended dose for phase II responded, for an overall response rate of 100% with 62% in complete remission. Expansion of CB-NK cells occurred as early as 3 days post infusion. The preliminary results of this study indicate high tolerability and activity. In summary, randomized clinical trials are needed to assess the long-term safety and efficacy of these novel cellular therapies.

## 8 Treatment summary

In the frontline setting, patients are treated according to age. For early-stage HL, we generally recommend a combined modality approach with chemotherapy (ABVD and/or eBEACOPP) and radiation, or risk-adapted PET-guided approach. Patients aged <60 years with advanced stage disease and no significant comorbidities, we recommend AAVD. Young women with disease in the thorax who may receive radiation exposure to breast tissue, we recommend chemotherapy alone approaches. In patients aged >60 years, the intensity of therapies is based on comorbidities. In the relapsed setting, chemoimmunotherapeutic or chemotherapy-free approaches can be used. AHSCT is considered the standard treatment for patients with r/r cHL and among those who experience r/r cHL after HDC/AHSCT, single agent and combinations with novel agents including BV and/or PD1 has demonstrated substantial efficacy and favorable toxicity. For a select population of patients, alloHSCT should be considered. Ongoing trials are evaluating the efficacy of CAR T-cells therapy in r/r cHL.

## 9 Conclusion

Classical Hodgkin lymphoma is a rare lymphoma, and while the majority of patients will respond to frontline therapy, up to 30% of patients may experience relapsed or refractory disease. The therapeutic approach to each patient depends on clinical prognostic factors, comorbidities and toxicity profile. Undeniably high dose chemotherapy and AHSCT remains the standard consolidation for patients whose disease responds to salvage systemic therapy, however, treatment options have dramatically changed over the years. The integration of novel therapies with standard regimen has achieved higher response rates and durable benefits with tolerable toxicity. Brentuximab vedotin and checkpoint inhibitors have shown survival benefits in patients whose HL has relapsed after AHSCT. More recently, CAR T-cell therapy has demonstrated exceptional response rates and safety, however longer follow up is required to confirm durability of responses. The new era of immune-chemotherapy and targeted agents continues to provide significant clinical benefits, although there is still a subset of patients with refractory disease who do not experience durable long-term responses with current treatment paradigms, and this remains a significant unmet need.

## Author contributions

The authors confirm contribution to the paper as follows: study conception and design: SA and FU; data collection: FU, DD, NO, and OO; draft manuscript preparation: all authors.
